# Solid Phase Biosensors for Arsenic or Cadmium Composed of A *trans* Factor and *cis* Element Complex

**DOI:** 10.3390/s111110063

**Published:** 2011-10-25

**Authors:** Mohammad Shohel Rana Siddiki, Yasunari Kawakami, Shunsaku Ueda, Isamu Maeda

**Affiliations:** 1 United Graduate School of Agricultural Science, Tokyo University of Agriculture and Technology, 3-5-8 Saiwaicho, Fuchu 183-8509, Japan; E-Mails: msrsiddiki@yahoo.com (M.S.R.S.); uedashun@cc.utsunomiya-u.ac.jp (S.U.); 2 Faculty of Agriculture, Utsunomiya University, 350 Minemachi, Utsunomiya 321-8505, Japan; E-Mail: kaaaaa25@yahoo.co.jp (Y.K.)

**Keywords:** biosensor, ArsR, CadC, GFP, metals, *cis* element, *trans* factor, lyophilization

## Abstract

The presence of toxic metals in drinking water has hazardous effects on human health. This study was conducted to develop GFP-based-metal-binding biosensors for on-site assay of toxic metal ions. GFP-tagged ArsR and CadC proteins bound to a *cis* element, and lost the capability of binding to it in their As- and Cd-binding conformational states, respectively. Water samples containing toxic metals were incubated on a complex of GFP-tagged ArsR or CadC and *cis* element which was immobilized on a solid surface. Metal concentrations were quantified with fluorescence intensity of the metal-binding states released from the *cis* element. Fluorescence intensity obtained with the assay significantly increased with increasing concentrations of toxic metals. Detection limits of 1 μg/L for Cd(II) and 5 μg/L for As(III) in purified water and 10 µg/L for Cd(II) and As(III) in tap water and bottled mineral water were achieved by measurement with a battery-powered portable fluorometer after 15-min and 30-min incubation, respectively. A complex of freeze dried GFP-tagged ArsR or CadC binding to *cis* element was stable at 4 °C and responded to 5 μg/L As(III) or Cd(II). The solid phase biosensors are sensitive, less time-consuming, portable, and could offer a protocol for on-site evaluation of the toxic metals in drinking water.

## 1. Introduction

Humans are widely exposed to various environmental pollutants which cause major health concerns in the developing World. The detection and monitoring of those pollutants in water are very important for human safety and security [1]. Some such threats to human health are sometimes associated with exposure to toxic metals like lead, cadmium, mercury and arsenic through contamination of drinking water and their entrance to the food chain [2–4]. The WHO drinking water guidelines recommend 3 μg/L for cadmium and 10 μg/L for arsenic along with the national regulatory standard (e.g., 50 μg/L As in India and Bangladesh) [5,6]. Long term exposure to arsenic through groundwater has been recognized as a major public health hazard in developing countries where unmonitored groundwater is the primary source of drinking water [7].

One effective way to reduce such risks is monitoring of toxic metals in drinking water. Standard laboratory-based traditional analytical methods such as atomic absorption spectrometry (AAS) and atomic fluorescence spectrometry, neutron activation analysis, inductively coupled plasma (ICP) techniques, and high-pressure liquid chromatography are routinely used for metal quantification, but those traditional methods require expensive and bulky laboratory equipment, analytical expertise, sample transportation and pre-treatment(s) [8–10].Therefore, field-applicable, simple and inexpensive detection methods need to be developed to compensate for the shortcomings of traditional laboratory-based techniques. Biosensors have already proved to be simple and cost-effective tools for quantification of toxic metals [11]. Biosensors are integrated devices that consist of a biological molecule for recognition in direct contact with a transduction element [1].

**Figure 1.**
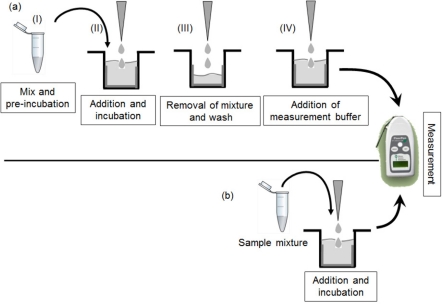
Comparison of the assay procedures of toxic metals using GFP-tagged *trans* factor and *cis* element in microplate wells. The previously developed procedure is composed of (I) mixing and 15-min pre-incubation of GFP-tagged *trans* factor with sample; (II) addition of the mixture to the well and 15-min incubation with immobilized *cis* element; (III) removal of the mixture and washing the well surface; and (IV) addition of measurement buffer (**a**). A newly developed procedure is composed of addition of sample mixture to the well and 15-min or 30-min incubation with GFP-tagged *trans* factor binding to immobilized *cis* element (**b**). Fluorescence of the supernatants can be measured with a handheld, battery-powered portable fluorometer.

The green fluorescent protein (GFP) has emerged as a powerful reporter molecule for monitoring gene expression, protein localization and protein-protein interaction [12]. It has been demonstrated that *in vitro* interaction takes place between a *trans* factor and a *cis* element of bacterial transcriptional switches [13]. The report indicates that a degree of the interaction can be quantified by fusing GFP to the C-terminus of a *trans* factor and immobilizing a *cis* element on solid surface, and the fluorescent intensity decreases with an increase in toxic metal concentrations. In the assay, *E. coli* cell lysates containing ArsR-GFP or CadC-GFP were pre-incubated with As(III) or Cd(II) solution, loaded into wells, on which *ars* promoter-*ars* operator (*P*_ars_-*O*_ars_) or *cad* promoter-*cad* operator (*P*_cad_-*O*_cad_) were immobilized, respectively, incubated for 15 min and removed from the wells (Figure 1(a)). Then, the wells were washed off once by phosphate buffer to remove extra proteins and filled with measurement buffer to dissociate proteins from the *cis* element. The supernatant was removed from the wells and poured into a measurement vial. Finally, fluorescence intensities of ArsR-GFP or CadC-GFP of the supernatant in the measurement vial were measured. Thus, the process required many steps for measurement of metals, therefore lessening of the number of steps in the procedure is essential for easy application of the biosensors to field tests (Figure 1(b)). To minimize storage and transportation costs, lyophilization is one of the most popular techniques for preserving proteins under normal refrigeration conditions, compared to −20 °C or −80 °C. Moreover, in the previous study [13], lyophilized lysates had to be rehydrated by addition of purified water before use.

**Figure 2.**
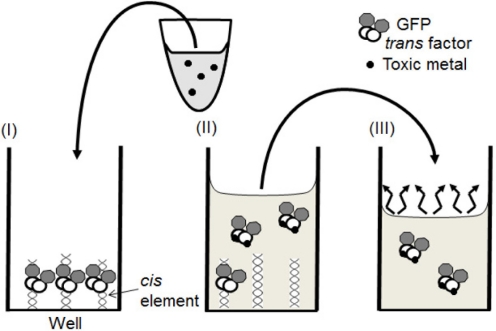
Expected association/dissociation statuses between GFP-tagged *trans* factor and *cis* element or metal. A sample mixture is loaded into (I), a well in which a biosensor composed of GFP-tagged *trans* factor binding to an immobilized *cis* element is constructed. Then; (II) the mixture is incubated in the well; and (III) the supernatant, which might contain the GFP-tagged *trans* factor dissociated from the *cis* element, was transferred to a well of another microplate or a glass vessel to measure its fluorescence intensity.

The aim of this work is to develop solid phase biosensors, in which water samples could be directly added to a complex of GFP-tagged *trans* factor and immobilized *cis* element and toxic metals could be quantified with fluorescence of GFP-tagged *trans* factor released from the *cis* element (Figure 2). As, in the new method the number of steps in the assay procedure are reduced and the complex is rehydrated by direct addition of sample, these solid phase biosensors could be advantageous for simple and on-site detection of toxic metals compared to traditional analytical methods.

## 2. Materials and Methods

### 2.1. Construction of Expression Vectors of Gene Encoding GFP-Tagged *trans* Factor

A specific reporter has been constructed in which the arsenic-binding regulatory protein gene, *ars*R originated from *Escherichia coli* K12 DNA, and the cadmium-binding regulatory protein gene, *cadC* from *Staphylococcus aureus* NCTC50581 plasmid pI258 have been fused to the structural gene for green fluorescent protein. A gene encoding green fluorescent protein from the marine species *Aequorea coerulescens* (excitation maximum: 475 nm; emission maximum: 505 nm) was excised from pAcGFP1 (Takara, Shiga, Japan), and ligated to an expression vector, pET-3a (Novagen-Merck, Darmstadt, Germany), to construct expression vectors of genes encoding ArsR-GFP or CadC-GFP, as described previously [13]. Cells of *Escherichia coli* BL21 (DE3) pLysS were transformed with the expression vectors.

### 2.2. Preparation of GFP-Tagged *trans* Factor

Cells of recombinant *E. coli* were grown in a Sakaguchi flask containing 300 mL of autoclaved Luria-Bertani (LB) medium supplemented with 50 μg/mL ampicillin and 34 μg/mL chloramphenicol at 25 °C for 24 h in an orbital shaker at 140 rpm. The cells were harvested from a culture containing 2 × 10^9^ cells/mL by centrifugation at 4 °C, and washed twice with 50 mM Tris-HCl buffer pH 7.4. The cells were resuspended into 4 mL TG buffer (50 mM Tris-HCl pH 7.4, 15% (v/v) glycerol) and frozen at −80 °C at least 1 h. After thawing, cells in a glass vial placed on ice were disrupted for 5 min with an ultrasonic disruptor equipped with a microtip probe (UD-201, Tomy, Tokyo). Sonication was repeated four times with 5-min interval. After centrifugation at 16,000 ×*g* for 15 min at 4 °C to remove cell debris, the lysate was divided into small aliquots and stored at −80 °C. The approximate concentration of GFP-tagged *trans* factor in cell lysate was calculated from fluorescence intensity [13].

### 2.3. Preparation of Promoter-Operator DNA and Its Immobilization on Microplate Well

**Figure 3.**
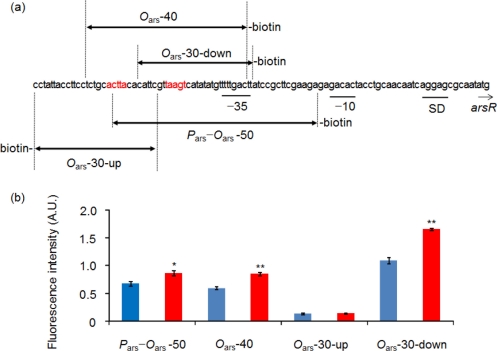
Nucleotide sequences of the oligonucleotides containing *O*_ars_ (**a**) and effects of their sequence difference on the biosensor response to As(III) (**b**). The ArsR binding sites are shown in red (a). The putative promoter sequences at the −35 and −10 recognition sites and the Shine-Dalgarno sequence (SD) are underlined. The oligonucleotide ends, to which biotin was bound, was shown. Fluorescence was measured with fluororeader (b). Average fluorescence intensities are shown with blue bars for no addition of As(III) and red bars for 100 μg/L As(III) in purified water. Statistical significance was shown as * *p* < 0.01; ** *p* < 0.001.

Oligonucleotide sequences for *P*_ars_-*O*_ars_-50 [13] and DNA fragments containing *O*_ars_ designed in this study were shown in Figure 3(a). The sequence for *P*_cad_-*O*_cad_-50 was indicated previously [13]. Twenty-five picomoles per 100 μL double-stranded DNA fragments modified with biotin at the 5′ or 3′ end in 25 mM Tris-HCl buffer (pH 7.4) were poured and immobilized onto Reacti-bind streptavidin-coated high binding capacity black 96-well microplate wells (Thermo Fisher Scientific, Yokohama, Japan) as described previously [13]. After immobilization, excess unbound DNA was rinsed off three times by 25 mM Tris-HCl buffer pH 7.4.

### 2.4. Preparation of the Solid Surfaces Including GFP-Tagged *trans* Factor

A solid surface was prepared by contacting ArsR-GFP to *O*_ars_-containing immobilized oligonucleotides. A mixture containing ArsR-GFP was prepared at final concentrations of 50 mM potassium phosphate buffer pH 7.4 (KPB), 50 μg/mL salmon sperm DNA, 40 mM NaCl and approximately 20 μg/mL ArsR-GFP. On the other hand, another solid surface was prepared by contacting CadC-GFP to immobilized *P*_cad_-*O*_cad_-50. A mixture containing CadC-GFP was prepared with a similar composition as that described in case of ArsR-GFP, except that 50 mM Tris-HCl buffer pH 7.4, instead of 50 mM KPB, was used. One hundred microliters of ArsR-GFP or CadC-GFP mixture were poured to each well in which oligonucleotide was immobilized, and incubated for 15 min. Free proteins were once washed off with 200 μL KP-T buffer (10 mM potassium phosphate buffer pH 6.0, 0.05% (w/v) Tween20).

### 2.5. Sample Preparation and Assay Procedure

As(III) and Cd(II) solutions were prepared by dissolving 98% NaAsO_2_ and CdCl_2_·2.5H_2_O (both from Sigma-Aldrich) in ultrapure water (Simplicity UV, Milipore-Japan, Tokyo), bottled natural mineral water, or tap water collected at Utsunomiya University. For the As(III) assay, 93.5 volumes of sample was mixed with 5 volumes of 1 M KPB pH 6.7, 0.5 volumes of 10 mg/mL salmon sperm DNA and 1 volume of 4 M NaCl (final concentrations; 50 mM KPB, 50 μg/mL salmon sperm DNA, 40 mM NaCl). For the Cd(II) assay, the sample was mixed with a solution of similar composition with the exception that 1 M Tris-HCl pH 7.9 was used instead of 1 M KPB. Ninety microliters of sample mixture were added to each well in which either of the solid surfaces was prepared, and incubated for 30 min in As(III) assay and for 15 min in Cd(II) assay, respectively, with orbital shaking at 120 rpm to release the GFP-tagged *trans* factor binding to metal from immobilized *cis* element. The supernatants were transferred to wells in another black plate when fluorescence intensity was measured with a microplate fluororeader at excitation/emission wavelengths of 490/530 nm (MTP-601, Hitachi High Technologies, Tokyo). The supernatants were transferred to glass vials when fluorescence intensity was measured with a handheld, battery-powered portable fluorometer (GFP-pen GFP 100, Photon Systems Instruments, Brno, Czech Republic). Student’s *t*-test was used to evaluate probability between two groups including data obtained with ultrapure water.

### 2.6. Lyophilization of the Solid Surface

For lyophilization, solid surfaces on wells after removal of washing buffer were frozen at −80 °C for at least an hour. Then, the solid surfaces were lyophilized with a freeze dryer (VD-500F, Taitec, Saitama, Japan) and stored in a refrigerator for 24 h. The lyophilized solid surfaces were evaluated by directly loading the sample mixtures.

## 3. Results and Discussion

In this study, solid surface biosensors comprising ArsR-GFP binding to the *O*_ars_ sequences or CadC-GFP binding to the *O*_cad_ sequences were used to analyze As(III) and Cd(II) solutions, respectively.

### 3.1. Development of Solid Phase Biosensors for Arsenic Detection

A solid phase biosensor constructed with *P*_ars_-*O*_ars_-50 and ArsR-GFP did not show a marked increase in the fluorescence intensity obtained with 100 μg/L As(III) (Figure 3(b)). Therefore, other solid phase biosensors were constructed using the oligonucleotides containing the different regions of *O*_ars_ and their responses to 100 μg/L As(III) were evaluated with a fluororeader. The biosensor comprising ArsR-GFP and *O*_ars_-30-down showed the highest fluorescence intensity, and the difference between the fluorescence intensities obtained with and without As(III) was more marked, compared to the differences in the biosensors comprising *O*_ars_-40 or *P*_ars_-*O*_ars_-50 (Figure 3(b)). On the other hand, only background levels of fluorescence were detected in the biosensor comprising *O*_ars_-30-up, regardless of the presence or absence of As(III). From the result, higher capacity of the *O*_ars_-30-down-immobilized solid surface for ArsR-GFP can be expected. Therefore, *O*_ars_-30-down was selected for the following experiments.

**Figure 4.**
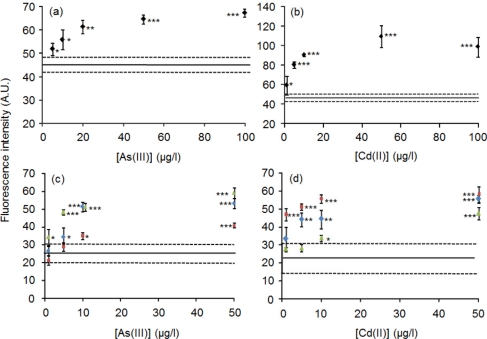
Dose-response relationships between As(III) (**a**,**c**) or Cd(II) (**b**,**d**) concentration and fluorescence intensity of the biosensor compo ed of ArsR-GFP and *O*_ars_*-*30-down (a,c) or the biosensor composed of CadC-GFP and *P*_cad_-*O*_cad_-50 (b,d). Average fluorescence intensities are shown with black symbols for ultrapure water, blue symbols for tap water, red for mineral water brand A, and green for mineral water brand B. Fluorescence was measured with fluorometer. A solid line and two broken lines show a mean ± SD of data obtained with water without addition of toxic metal. Statistical significance was shown as * *p* < 0.05; ** *p* < 0.01; *** *p <* 0.001.

Significant increases of fluorescence in response to 100 μg/L As(III) were observed with both fluororeader (*p* < 0.001) and fluorometer (*p* < 0.01) (data not shown). Therefore, a dose-dependent relationship between As(III) concentration and fluorescence intensity was investigated with the fluorometer.. A dose-dependent increase of fluorescence was observed and a detection limit of 5 μg/L for As(III) was achieved (Figure 4(a)). The biosensor comprising ArsR-GFP and *O*_ars_-30-down also responded in the detection of As(III) in drinking water. The responses of the solid phase biosensor to As(III) were analyzed using tap water and two types of bottled natural mineral water. A method detection limit of 10 μg/L As(III) was obtained with fluorometer (*p* < 0.01) when the fluorescence intensities were compared with the average intensity of all water without addition of As(III) (Figure 4(c)). Significant differences were also observed at 10 μg/L based on comparison within individual water. Thus, the optimized solid phase biosensor could offer a simple protocol for on-site screening of As(III)-containing drinking water

### 3.2. Detection of Cd(II) with the Solid Phase Biosensor

It was examined in the solid phase biosensor whether dissociation of CadC-GFP from *P*_cad_-*O*_cad_-50 is enhanced by Cd(II) within 15 min or not. Sample mixtures containing different concentrations of Cd(II) were poured into the wells, in which the solid phase biosensor was constructed. The fluorescence intensities measured with fluorometer increased with the increases in Cd(II) concentration up to 50 μg/L (Figure 4(b)). A detection limit of 1 μg/L Cd(II) was obtained using ultrapure water. The significant responses of CadC-GFP to Cd(II) in tap water and mineral water brand A were 5 μg/L (*p* < 0.01) and 1 μg/L (*p* < 0.001) with comparison to the average of all kinds of water tested (Figure 4(d)). A significance difference was found at 10 μg/L (*p* < 0.05) using mineral water brand B. Significant differences were observed at 5 μg/L based on comparison within individual water. Therefore, the solid phase biosensor is also available for monitoring of Cd(II) in drinking water.

Kawakami *et al*. [13] reported a detection limit of 1 μg/L for Cd(II) in the previous assay procedure, which is composed of 15-min pre-incubation of cell lysates containing CadC-GFP with samples, 15-min incubation of the mixtures in wells where *P*_cad_-*O*_cad_-50 was immobilized, and measurement of fluorescence, as shown in Figure 1(a). Therefore, it would be possible to conclude that the solid phase biosensor reduces the assay time and lessens the steps of assay procedure in the previously reported CadC-GFP biosensor while keeping the sensitivity to Cd(II).

### 3.3. Responsiveness of the Lyophilized Solid Phase Biosensors

**Figure 5.**
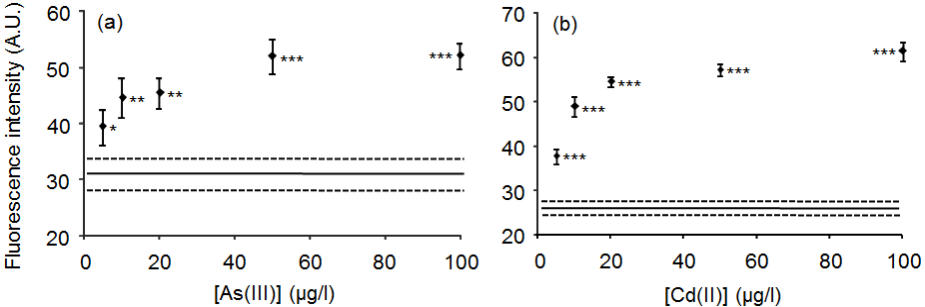
Dose-response relationships between As(III) (**a**) or Cd(II) (**b**) concentration and fluorescence intensity of the lyophilized solid phase biosensors. Fluorescence was measured with fluorometer. A solid line and two broken lines show a mean ± SD of data obtained with ultrapure water. Statistical significance was shown as * *p* < 0.05; ** *p* < 0.01; *** *p <* 0.001.

The solid phase biosensors were lyophilized to enable preservation in the refrigerator. A microplate modified with lyophilized ArsR-GFP binding to *O*_ars_-30-down or lyophilized CadC-GFP binding to *P*_cad_-*O*_cad_-50 was used to evaluate fluorescence intensities obtained with different concentrations of As(III) or Cd(II), respectively. Detection limits for As(III) and Cd(II) were 5 μg/L with a fluorometer (Figure 5(a,b)). Therefore, the lyophilized solid phase biosensors were preserved stably at 4 °C without lyoprotectants. The dose-dependent increase of fluorescence by the lyophilized biosensors was more marked in Cd(II) than in As(III). Lyophilization increases shelf life, flexibility and stability of the fluorescence protein [14,15]. Therefore, lyophilization is required to produce the stable solid phase biosensors available for practical use.

### 3.4. Features of the Solid Phase Biosensors

*Escherichia coli* ArsR [11] and *Staphylococcus aureus* CadC [16] lose binding capability to *O*_ars_ or *O*_cad_ in their metal-binding conformations, respectively [11,16–19]. It is worth to note that in the previously developed biosensors, the association process between *trans* factor and *cis* element was evaluated [13] whereas, in the solid phase biosensors, their dissociation process was evaluated. Another important fact demonstrated in this study is that the binding capacity and association/dissociation ratio of ArsR-GFP in response to As(III) are markedly affected by the nucleotide sequences chosen from the promoter–operator region. The response of the solid phase biosensor to As(III) was successively improved by choosing *O*_ars_-30-down.

This study showed that the newly developed solid phase biosensors respond to As(III) and Cd(II). However, besides the specific toxic metals, the biosensors must respond to Sb(III), Pb(II) and Zn(II) because the specificities of ArsR-GFP to Sb(III) and CadC-GFP to Pb(II), Zn(II) and Sb(III) have been demonstrated [13]. The generally recognized advantages of metal-monitoring biosensors over traditional methods such as AAS and ICP are their capability of on-site measurement, low cost and easy manipulation. In this study, solid phase biosensors with easy manipulation were successively developed while maintaining the low detection limits (<10 μg/L) of the previously developed biosensors [13] in comparison with the detection limits of flame AAS (∼1 mg/L) [20,21], ICP-Atomic Emission Spectroscopy (30 μg/L) [22].

When the solid phase biosensors are compared with biosensors that utilize transcriptional switches to respond to analytes and to trigger signal transduction, their superior features can be highlighted. The solid phase biosensors elicit a significant response within 30 min for As(III) and 15 min for Cd(II), which are much shorter than the times required by whole-cell based biosensors whose responses usually take place within 2 to 3 h [22]. The biosensor based on *in vitro* reconstitution of the transcriptional switch has been reported [23]. In the assay, however, it takes 2 h to bind tetracycline-*Renilla* luciferase-tagged repressor protein (TetR-Rluc) on its repressor-operator site. In addition to this long assay time requirement, enzyme reactions of luciferase remaining on the wells is needed to obtain a luminescence signal, whereas, in the solid phase biosensors, the fluorescence signal is directly produced by GFP-tagged ArsR or CadC protein with an increase in toxic metal concentrations because dissociation from *cis* element becomes pronounced in the presence of As(III) or Cd(II). The capability of direct sample addition is another advantage of the solid phase biosensors. In comparison with the previously developed biosensors [13,23], the solid phase biosensors are advantageous in terms of the required assay time and protocol simplicity. Additionally, their ease of handling and storage are worth mention as the whole elements of biosensor can be preserved in a same package without lyoprotectants under normal refrigeration conditions, and can be rehydrated by the direct addition of sample.

Among environmentally found heterogeneous elements, the hardness of water may weaken the fluorescence intensity of CadC-GFP to Cd(II) because the tendency was more marked in very hard water brand B (203 mg/L as CaCO_3_) than in soft water, tap water (56.1 mg/L as CaCO_3_) and brand A (42.9 mg/L as CaCO_3_). Contrary to this tendency, higher fluorescence responses of ArsR-GFP to As(III) were found in mineral water brand B. The opposite effects might be caused due to different behavior of the *trans* factors (CadC and ArsR) in very hard water and/or different reactivities of the buffers (Tris-HCl and KPB) to minerals. Therefore, in very hard water, dissociation of CadC-GFP from the solid phase might be partially hampered, resulting in the lower fluorescence values. In case of CadC-GFP, by taking an adequate control adjusted to the hardness of water, the solid phase biosensors can be provided as a common tool for monitoring of Cd(II) in drinking water. Thus, it was demonstrated that the developed solid phase biosensors offer a simple operating procedure and have the portability, stability and sensitivity which are necessary for monitoring of toxic metals in drinking water.

## 4. Conclusions

The dissociation process of the complexes composed of GFP-tagged *trans* factor and immobilized *cis* element could be applied to a simple and rapid protocol to detect As(III) or Cd(II) in drinking water. Prepared protein–DNA complexes are more preferable for lyophilization in a same package and can be stored under normal refrigeration conditions after manufacturing a sensor kit for the toxic metals with detection limits of <5 μg/L based on ultrapure water and <10 μg/L based on tap water and bottled mineral water. The manufactured kits could be handled just by adding samples directly to microplate wells, and results would be obtained with 30-min and 5-min incubation for As(III) and Cd(II), respectively. Another big advantage is its portability because the handheld battery-powered fluorometer can be used anywhere to measure the fluorescence intensity that reflects the toxic metal concentrations.
